# Threat of Sexual Disqualification: The Consequences of Erectile Dysfunction and Other Sexual Changes for Gay and Bisexual Men With Prostate Cancer

**DOI:** 10.1007/s10508-016-0728-0

**Published:** 2016-04-21

**Authors:** Jane M. Ussher, Janette Perz, Duncan Rose, Gary W. Dowsett, Suzanne Chambers, Scott Williams, Ian Davis, David Latini

**Affiliations:** 1 Centre for Health Research, School of Medicine, Western Sydney University, Sydney, NSW 2751 Australia; 2 Australian Research Centre in Sex Health and Society, La Trobe University, Melbourne, VIC Australia; 3 Menzies Health Institute, Griffith University, Nathan, QLD Australia; 4 Peter MacCallum Cancer Centre, Melbourne, VIC Australia; 5 Eastern Health Clinical School, Monash University, Melbourne, VIC Australia; 6 Psychiatry and Behavioral Sciences, Baylor College of Medicine, Houston, TX USA; 7 Australian and New Zealand Urogenital and Prostate Cancer Trials Group, Camperdown, NSW Australia

**Keywords:** Prostate cancer, Gay and bisexual men, Erectile dysfunction, Sexual changes, Gay identity, Masculinity

## Abstract

Gay and bisexual (GB) men with prostate cancer (PCa) have been described as an “invisible diversity” in PCa research due to their lack of visibility, and absence of identification of their needs. This study examined the meaning and consequences of erectile dysfunction (ED) and other sexual changes in 124 GB men with PCa and 21 male partners, through an on-line survey. A sub-sample of 46 men with PCa and seven partners also took part in a one-to-one interview. ED was reported by 72 % of survey respondents, associated with reports of emotional distress, negative impact on gay identities, and feelings of sexual disqualification. Other sexual concerns included loss of libido, climacturia, loss of sensitivity or pain during anal sex, non-ejaculatory orgasms, and reduced penis size. Many of these changes have particular significance in the context of gay sex and gay identities, and can result in feelings of exclusion from a sexual community central to GB men’s lives. However, a number of men were reconciled to sexual changes, did not experience a challenge to identity, and engaged in sexual re-negotiation. The nature of GB relationships, wherein many men are single, engage in casual sex, or have concurrent partners, influenced experiences of distress, identity, and renegotiation. It is concluded that researchers and clinicians need to be aware of the meaning and consequences of sexual changes for GB men when designing studies to examine the impact of PCa on men’s sexuality, advising GB men of the sexual consequences of PCa, and providing information and support to ameliorate sexual changes.

## Introduction

Prostate cancer (PCa) is the most common non-cutaneous cancer affecting men in the West, and the second most common cause of cancer-related death (Siegel, Miller, & Jemal, 2015). While PCa treatments have had a dramatic impact on 5 years survival rates, which currently stand at between 84 and 92 % (Australian Institute of Health and Welfare, 2012; Cancer Research UK, 2015), such treatments can have a long-term impact on men’s sexual functioning. This includes erectile difficulties, non-ejaculatory orgasms, and decreases in desire and sexual satisfaction (Chung & Brock, 2013), often accompanied by bowel and urinary incontinence (Daniel & Haddow, 2011). These sexual changes have been associated with anxiety and depression (Perz, Ussher, & Gilbert, 2014), as well as threats to masculine identity (Zaider, Manne, Nelson, Mulhall, & Kissane, 2012). However, until recently, most research examining the impact of PCa on men’s sexuality has focused on the ability to achieve and maintain an erection for penile-vaginal penetration (Wittman et al., 2009), assuming that men are in long-term, monogamous, heterosexual relationships, and implicitly excluding the experiences of single and gay men (Asencio, Blank, Descartes, & Crawford, 2009).

Of the 19,993 new cases of PCa reported in Australia in 2011 (Australian Institute of Health and Welfare, 2015), conservative estimates based on the recorded percentage of gay men in the population, suggest that three to five percent are gay men (Susman, 2011). This means that 650–1000 Australian gay men are diagnosed with PCa each year, and that 6500–10,000 are living with the disease. Comparable estimates for the U.S. are 5000 gay men diagnosed every year, with over 50,000 gay men living after PCa treatment (Blank, 2005). Bisexual men, and heterosexually identified men who also have sex with men, are not included in these population estimates, suggesting that the proportion of all men who have sex with men (MSM) living with PCa is much higher. There has been calls for health promotion and education to acknowledge that gay and bisexual (GB) men with PCa may experience health concerns differently to heterosexual men (Filiault, Drummond, & Riggs, 2009; Filiault, Drummond, & Smith, 2008; Galbraith & Crighton, 2008). However, recent reviews of PCa educational resources and lesbian, gay, bisexual, and transgender (LGBT) primary care guidelines report a dearth of such information (Duncan, Watson, Westle, Mitchell, & Dowsett, 2011; McNair & Hegarty, 2010), with a few notable exceptions (Buchting et al., 2015; Wong et al., 2013), and there is an absence of empirical research to inform its future development. This has led to GB men with PCa being described as an “invisible diversity” (Blank, 2005), or a “hidden population” (Filiault et al., 2008).

There is some evidence from recent survey-based research that gay men with PCa report significantly greater difficulties in relation to sexual functioning (Motofei, Rowland, Popa, Kreienkamp, & Paunica, 2011), urinary, bowel and mental functioning (Hart et al., 2014; Ussher et al., 2016), and ejaculatory bother (Wassersug, Lyons, Duncan, Dowsett, & Pitts, 2013) in comparison with heterosexual men. Gay men with PCa have also been shown to report worse physical symptoms and greater fear of PCa recurrence compared with heterosexual norms (Hart et al., 2014), as well as lower satisfaction with PCa health care (Torbit, Albiani, Crangle, Latini, & Hart, 2015). However, quantitative research in this field has been limited by comparing GB men with population norms, rather than a comparable sample of heterosexual men (e.g., Hart et al., 2014; Torbit et al., 2015), or utilizing small samples of GB men, thus precluding statistical analysis (e.g., Lee, Breau, & Eapen, 2013; Motofei et al., 2011). Qualitative research has suggested that gay men experience significant concerns about changes in their sexual well-being, relationships, and gay identity following diagnosis of PCa (Fergus, Gray, & Fitch, 2002; Filiault et al., 2009; Hartman et al., 2014). However, these findings are based on the accounts of small numbers of participants, or involve asking GB men who do not have PCa about their perceptions of concerns (Asencio et al., 2009).

The primary focus on the physical effects of cancer or cancer treatments on sexual functioning assumes that a man’s experience of sexuality is limited to its embodied dimensions, negating the influence of the social construction of sexuality and gender, and the ways in which men interpret and experience physical changes in the light of such social constructions (Gilbert et al., 2013). Constructions of sexuality and masculinity are highly interwoven, meaning that loss of sexual functioning poses a significant threat to manhood and masculinity (Arrington, 2003; Bokhour, Clark, Inui, Silliman, & Talcott, 2001; Fergus et al., 2002); however, there is a dearth of research on the potential impact of PCa on the identity or masculinity of GB men. In one qualitative study examining knowledge about PCa in healthy gay men (Asencio et al., 2009), participants speculated that gay men would be more able than heterosexual men to come to terms with challenges to their masculinity, because of being part of a sexual minority. Conversely, a quantitative study reported lower rates of masculine self-esteem in GB men with PCa in comparison with heterosexual men (Ussher et al., 2016). Further research is needed to examine this issue. It has also been posited that gay men may ascribe different priorities and meanings to sexual changes after PCa (Thomas, 2012), including the importance of the prostate as a site of pleasure during anal sex; the significance of visible ejaculate for “semen exchange” during sex; the need for a firmer erection for anal sex in comparison with vaginal sex; and the consequences of anal discomfort and incontinence for receptive partners (Filiault et al., 2008). However, these concerns have been described as “speculative,” with “future research needed to ascertain the impact of PCa on the lives of gay men” (Filiault et al., 2008, p. 328).

Gay men are more likely than heterosexual men to be single, with only 42.9 % of Australian gay men reporting being partnered in a recent study (Pitts, Smith, Mitchell, & Patel, 2006). However, a substantial proportion of GB men engage in casual sexual relationships (Liau, Millett, & Marks, 2006), with casual or concurrent sexual relationships also reported by approximately 50 % of those who are partnered (Wassersug et al., 2013). Gay men are less likely to cohabit with a long-term partner than heterosexual men (Blank, 2005), with higher rates of living alone reported by older gay men (Wassersug et al., 2013). There is an absence of knowledge about the experience of PCa within the open relationships in which some GB men engage, little knowledge about the PCa experience of single GB men (Blank, 2005; Filiault et al., 2008), and no research examining the experience of male partners of GB men with PCa, with the exception of qualitative case studies (Filiault et al., 2008; Hartman et al., 2014).

The aim of this study was to address these gaps and inconsistences in the research literature by examining the meaning and consequences of sexual changes following PCa for GB men, using a mixed method research design. The following research questions were addressed: Which sexual changes following PCa are of concern to GB men with PCa and male partners? What are the meanings and perceived consequences ascribed to such changes?

## Method

### Participants

A combination of on-line survey and one-to-one interviews was used to examine the meaning and consequences of sexual changes after PCa for GB men and male partners. The survey provided information on the percentage of a broad sample of men with PCa reporting specific sexual changes; interviews with a sub-sample of survey respondents and partners facilitated in-depth examination of the subjective interpretation, meanings, and perceived consequences of such changes.

A total of 124 GB men who currently have, or have had, PCa, and 21 male partners of men with PCa participated in the study, part of a larger program of mixed methods research examining sexual well-being and quality of life after PCa in GB men and their partners, in comparison with heterosexual men (Rose, Ussher, & Perz, 2016; Ussher et al., 2016). The average age of men with PCa was 64.25 years, with partners 55.57 years; PCa was diagnosed 5 years previously on average, resulting in a range of treatments, with the majority of participants currently being monitored post-treatment. Full demographic details are presented in Table 1. Participants were primarily recruited within Australia, with a minority recruited from the U.S. and the U.K., through a range of recruitment strategies: distribution of an information sheet by collaborating urology and general practice clinicians, cancer research databases, GB-specific and general PCa cancer support groups in rural and urban locations, and GB community organizations; advertisement for the study and link to the information sheet posted on GB social media, and on electronic listserves targeting PCa survivors. After reading the information sheet describing the research team, the purposes of the research, and details regarding participation, participants completed an on-line survey examining their experiences of sexuality, intimate relationships, and psychological well-being post-cancer. At the end of the survey, participants indicated whether they would like to be considered to take part in an interview to discuss changes post-cancer in more depth. Of the 62 % GB men who indicated a willingness to be interviewed, 53 took part in semi-structured interviews, 46 GB men with PCa and 7 partners. Participants with PCa were purposively selected for an interview to ensure a broad sampling frame across age-groups, sexual orientations (gay/bisexual), relationship contexts (single/partnered; exclusive/non-exclusive), and experiences with prostate cancer (e.g., years since diagnosis, self-reported severity of sexual changes). All of the male partners who volunteered for interview were interviewed. Demographics and survey responses from the interview sample and the full sample were compared to determine representativeness, and there was no significant difference. Ethical approval was granted by Western Sydney University Human Research Ethics Committee and the ethics committees of participating community organizations, and all participants gave specific consent.

**Table 1:** Sociodemographic characteristics of gay/bisexual men with PCa and male partners of men with PCa

Variable	Patients (*N* = 124)		Partners (*N* = 21)	
	*n*	*M* (*SD*)	*n*	*M* (*SD*)
Age (in years)^a^	119	64.25 (8.18)	21	55.67 (9.04)
Years since diagnosis	115	5.904 (5.03)	20	3.35 (2.85)
	*n*	%	*n*	%
Sexuality				
Gay	99	81.15	19	90.48
Bisexual	23	18.95	2	9.52
Ethnicity				
Anglo-celtic	84	67.74	12	57.14
Other^c^	40	32.26	9	42.86
Country of residence				
Australia	85	69.67	14	66.67
USA	25	20.49	4	19.10
UK	10	8.20	1	4.80
NZ	1	0.82	2	9.60
Other	1	0.82	–	–
Employment status				
Fulltime/part-time	46	37.71	14	66.67
Retired/pension/social security	62	50.82	5	23.81
Other	14	11.48	2	9.52
Education				
High school	28	22.95	4	20.00
Tertiary diploma or trade certificate	25	20.49	5	25.00
University degree or higher	69	56.55	11	55.00
Relationship status				
Partnered (living/not living together)	60	49.58	–	–
Not in a relationship/other	61	50.41	–	–
Length of current relationship				
Less than 2 years	13	18.84	2	11.10
More than 2 years	56	81.16	16	88.90
Current casual sexual relationship				
Yes	49	39.84	6	28.57
No	74	60.16	15	71.43
Number of sexual partners in the last 6 months				
None	50	42.02	9	42.86
One	28	23.53	3	14.28
2 or more	41	34.45	7	33.33
Status of disease^b^				
No longer detectable	83	68.60	8	44.44
Receiving treatment	36	29.95	9	50.00
Other	2	1.65	1	5.55
Treatment received^b^				
Active surveillance	12	10.26	2	10.00
Radical prostatectomy	35	29.06	4	20.00
Robotic prostatectomy	21	17.95	6	30.00
Radiotherapy	15	11.97	1	5.00
Androgen ablation	2	1.71	–	–
Multiple treatments received	34	29.05	6	30.00

^a^Where* N* < 124 = missing data

^b^For partners, refers to status and treatment of the person with PCa

^c^“Other” includes African-American, South-American, South-East Asian, Middle East, each less than 2.4 %

### Measures and Procedure

A number of GB community organizations, GB prostate cancer survivors, and gay prostate cancer support group leaders provided advice on the development of the research protocol, survey, and interview questions. The survey items used in this analysis consisting of a series of closed- and open-ended questions examining the nature of sexual changes experienced by GB men with PCa. This included: items from the Expanded Prostate Cancer Index (EPIC)—Sexual Domain (Wei, Dunn, Litwin, Sandler, & Sanda, 2000): ability to achieve an erection, firmness of erections, frequency of erections, level of sexual desire, and how big a problem erectile ability or absence of desire has been; two items from the Changes in Sexual Functioning Questionnaire (CSFQ-M) (Keller, McGarvey, & Clayton, 2006): overall enjoyment in sex life (now and before cancer), and ability about ability to ejaculate; three items from Functional Assessment of Cancer Therapy-Prostate (FACT-P) (Esper et al., 1997): difficulty in urinating, increased frequency of urination, and problems with urination limiting activities; and three items developed for the present study: concern about ability to ejaculate, preferred role as insertive or receptive partner in anal sex, and discussion of sexual changes with health professionals. The open-ended survey questions asked for additional comments on how sexuality has changed since the onset of PCa; whether there have been any significant changes to relationships; and whether there were any other issues about PCa and sexuality that the participant would like to comment on.

One-to-one semi-structured telephone interviews, lasting approximately 1 h, were conducted to examine the subjective experience, meaning, and consequences of sexual changes following PCa, renegotiation of sexual practice in the context of casual and ongoing relationships, and support from health professionals. The majority of the interviews were undertaken by a gay man, with the exception of pilot interviews conducted by a woman interviewer. The interviews were conducted as an “extended conversation” (Rubin, 2005, p. 96), with the wording and formatting of questions used flexibly to suit the particular context and experience of the participants, drawing on responses to closed- and open-ended survey items. Interviewees were offered $25 gift card as a reimbursement for expenses. Sampling was discontinued when we had interviewed a cross section of men across the categories used for purposive sampling, outlined above.

### Analysis

Frequency data and percentages were collected for responses to the closed survey items. The analysis of open-ended survey responses and interviews was conducted using theoretical thematic analysis (Braun & Clarke, 2006). The style of analysis adopted was inductive with the development of themes being data driven, rather than based on pre-existing research or hypotheses. All of the interviews were audio-recorded and transcribed verbatim, with the resulting transcripts then read in conjunction with the audio recording, to verify for errors in transcription. A subset of the interviews were then independently read and reread by two of the authors to identify first-order concepts or codes, such as “concerns regarding sexual function,” “aging,” “sexual renegotiation,” “emotional consequences,” and “relationship context.” The entire dataset was then coded using NVivo, a computer package that facilitates organization of coded qualitative data. All of the coded data were then read through independently by two of the authors. Codes were then grouped into higher-order themes, a careful and recursive decision-making process, which involved checking for emerging patterns, for variability and consistency, and making judgments about which codes were similar and dissimilar, leading to the development of a thematic map of the data. In this final stage, a core category “meaning and consequences of sexual changes after PCa” was developed, which essentially linked all of the themes. In the presentation of the results below, we are reporting frequency of sexual changes drawing on the closed-ended survey responses of GB men with PCa, and the meaning and consequences of such changes drawing on the open-ended survey items and interviews with PCa survivors and partners. Pseudonyms were allocated to all participants, with information on age and identification as gay or bisexual provided after substantive qualitative responses. Status as a partner is also included for men who were partners.

## Results

### Erectile Dysfunction: “A Defining Moment in Life”

Loss of erectile functioning during the last 4 weeks was reported by 72 % of survey respondents, with 40 % of this group reporting that they could not achieve an erection and 32 % reporting only a partial erection; 18.7 % of the total sample reported that they could achieve an erection whenever they wanted; and 27 % reported an erection firm enough for anal intercourse (EPIC). Even when anal penetration was possible, the physiology of the rectum was reported to affect functioning, as Zachery (55, gay, partner) commented “with fucking he finds that my sphincter kind of deflates his erection.” Erectile functioning was also reported to affect masturbation, with Gareth (65, gay) saying that “it would be like playing with a piece of jelly” and Henry (59, gay) saying “it’s a really, really big effort to try and get the thing up, rubbing it…it’s just soft in your hand.” In open-ended survey responses and interviews, men described the emotional impact of these erectile changes, the impact on gay identity and masculinity, as well as the impact on sexual relationships, outlined below.

#### Emotional Impact of Erectile Changes: “You’re Not the Full Value” or Acceptance of Change

The majority of men (81 %) who reported loss or change in erectile functioning over the last 4 weeks rated it as a problem (EPIC). For 19 %, it was rated as a small or very small problem; for 61 %, it was rated as a moderate or big problem (EPIC), which had a “great emotional impact” and was experienced as “depressing,” “very difficult,” “an enormous loss,” or a cause of “great sadness.” For example, David (64, gay) said “I feel devastated; the erection functioning is a really emotional thing for me,” and Jonny (54, bisexual) said that “it’s quite a big thing for a man, especially for a younger man, at 49, not being able to have erections.” Many older participants said that as gay or bisexual men they expected to continue to have an active sexual life well into later life, with Sam (74, gay) commenting that “gay men tend to engage in sex for a longer period,” differentiating himself from heterosexual men in his age group, of whom he said “none of these men would be sexually active.” This expectation of continued sexual activity provides some explanation for the near uniformity in accounts of distress following erectile difficulties in older participants, for example: “it’s probably the most horrific thing that I’ve ever been through psychologically” (Finn, 69, gay); “it’s depressing. I feel like I’m sort of, pretty useless, because you’re not complete. You’re not the full value or something” (Clive, 70, gay).

Clive was not alone in experiencing a depleted sense of self following erectile loss; other men described themselves as “not feeling whole,” or feeling “cheated” of a core aspect of their masculinity. For example, Graham (74, gay) said “I am not the man I was, never will be,” and Finn (69, gay) told us “I’m no longer a man. I’ve got a cock that doesn’t work anymore.” The magnitude of this sense of loss is illustrated by Scott (59, gay), who said that “if I had the choice again, I would take my risks with the cancer, and not have the operation,” describing the loss of erections after robotic prostatectomy as “a defining moment in my life…the impact on my life as a gay male has been really profound, and in a negative sense.”

Long-term negative emotional reactions to ED were not inevitable, however. A number of men gave accounts of reconciling themselves to changes in sexual functioning, and developing fulfillment through other activities: “in life if my sexual function diminishes further, there are other aspects of life that will take over from that which will continue to make life satisfying and rewarding” (Alex, 69, gay); “I have developed more close and intimate relationships with men that don’t include sex” (Damon, 52, gay). Many men described engagement in creative pursuits, such as art, pottery, or music. These accounts demonstrate that for some men initial negative responses to ED can be replaced by acceptance and absorption in other pursuits.

#### Impact on Gay Identities: “Retired From the Gay Human Race” or Identity Re-evaluation

While erectile dysfunction (ED) is widely recognized to have a potential impact on masculine identities, it is the impact on *gay* identities that is identified in Scott’s account of changes to his life as a “gay male,” above. Many of the participants in this study emphasized the importance of sexual activity to their identity as a gay man, an identity that was threatened by ED. This is illustrated in the accounts below:

I think gay men are a lot more sexually aware, or I think part of our identity is that it’s about sex and our ability to function sexually, and I think we take a harder hit when it [ED] happens. (Rick, 59, gay)

I’m still a gay man but what that meant was, was suddenly quite different. Somehow being sexually active had always been a fundamental part of that identity, and that was then changed. And I suppose making sense of that was quite hard…my personal identity certainly went into a crisis. (Mark, 45, gay)

As a result of this “crisis” in identity, some men said that they did not “feel so good about being gay anymore” (Benjamin, 63, gay), felt “outside the sexual community” (Jason, 49, gay), or felt as if they had been “forcibly retired from the gay human race” (Scott, 59, gay). For men who had identified as gay in later life a double blow was reported—loss of sexual functioning and loss of further opportunity to explore recently discovered gay sex. For example, Clive told us that he had come out as gay when he was 50, and was diagnosed with advanced PCa 4 years later. He said that the “thrilling and scary” sexual exploration he had been engaged in “all sort of crashed before I’d even got there…the whole thing had gone before it even started,” and he felt “robbed of any opportunity [he] might have had.”

For many participants ED signified aging, with Mark (45, gay) saying, “I went from being a young gay man to feeling old” and Jack (59, gay) saying that “prostate cancer has made me feel older than I need to at this stage.” Being perceived as “old” was described as having a particular negative meaning for gay men. For example, Colin (68, gay) said that “it takes a certain amount of self-confidence and self-awareness and being comfortable with yourself, to be able to age and grow older within the gay community” and Alan (67, gay, partner) commented that “as an old gay man you’re not particularly marketable.” However, gay men who maintain sexual functioning may be able to resist being positioned as old. For example, Nick (age 66, bisexual) described himself as “really lucky” because he was “a very fit guy” with “a really nice body,” and could “fuck for 2 or 3 h or as long as a bottom [receptive partner] can take it,” as a result of penile injections. This made him feel “young” and attractive with “a lot of the younger guys attracted to me.”

In contrast, a number of men gave accounts of ED following PCa having no impact on their identity as gay men. For example, Alex (69, gay) said: “my worth of self, my functioning as a gay man, no, I don’t think it’s particularly impacted” and Euan said there was “no real change,” other than giving up his sexual position as a “top” or insertive partner in anal intercourse, which he felt he “had to make the best of.” Others suggested that they had re-evaluated their previous association of sexuality and gay identity, as evidenced by William’s account: “I suppose, as a gay person you identify yourself through your sexuality, which, you know, from where I’m sitting now, looks a bit silly” (67, gay). Finally, a number of men talked about their identity going through a transition following PCa, resulting in changes in how they operationalized their lives as gay men:

I had gone through my journey as a gay man of being sexual and being attractive and just having physical fun with other men, and it was like I’d come out the other side of that and that was gone, and it felt like, “well, you know, I’m still a gay man” but what that meant was, was suddenly quite different (Mark, 45, gay).

A number of men attributed their ED to aging, rather than PCa, or accepted ED because of their age. For example, Cameron (56, bisexual) said that “something has changed, and I choose to see that as a change in my age, more than a change due to the prostate cancer.” William (67, gay) described a friend who was the same age as himself who had ED and said “if I hadn’t had cancer, I probably would’ve been in the same position I am now anyway.” These accounts suggest that PCa related sexual dysfunction does not inevitably place men outside of the gay community and take away their sense of gay identity; it depends on whether individual men position erectile functioning as central to being a gay man, or an older man.

#### Relational Impact of Erectile Dysfunction: Threat of Sexual Disqualification or Sexual Renegotiation

ED was described by many participants as resulting in feeling “sexually inferior,” or “a eunuch,” leading to a sense of “disqualification in the sexual experience.” This demonstrates that ED can significantly influence GB men’s sexual and social interactions, with the consequences of dysfunction played out in a relational context. Many men gave accounts of avoiding sexual encounters with new or casual partners because of this, as Grant (72, gay) said “I don’t even like to think of trying to interest a new prospective partner in sex with me because of my limited ability to perform.” Mason (68, gay) said that he was “desperate” to be in a relationship but would not feel “worthy” and he was “worried that will affect my ability to find a partner.”

For men who took an insertive role in anal intercourse, the inability to achieve or maintain an erection had the potential to influence their sexual engagement with others significantly, often leading to the use of medical aids, or sexual “reinvention.” For example, Finn (69, gay) said “It was like someone taking away all your toys. Where you could just rock up with a guy and do whatever you like with him, and no longer could you do that…. I had to reinvent myself and that was very difficult.” Finn’s reinvention involved developing “an ability to perfect foreplay.” For some, the consequence was cessation of sex. For example, Scott (59, gay) described himself as having been “fortunate to have a bit of a following” where regular partners knew what they could expect “in terms of satisfaction”:

When I had the prostatectomy, five years ago now that’s completely changed and since then I’ve become a basically inactive gay male without the sex part unless it’s assisted through injections, which I really dislike because I don’t think any guy likes sticking needles into their dick.

This account demonstrates the social nature of erectile functioning, where the “following” Scott previously had as a “normally active gay male” who could guarantee sexual satisfaction to his partners has now gone. This resulted in social and sexual isolation.

For men who took a receptive role in anal intercourse, absence of erection could also be problematic, as Mark (45, gay) explained: “partners would comment, ‘Aren’t you turned on, aren’t you into this, don’t you want to do this?’.” Mark’s account illustrates the fact that erections have significance within gay men’s sexual encounters beyond the act of anal penetration. As Aaron (59, gay) commented, “erections are important, but they’re important visually,” with an erect penis signifying desire and pleasure: “it’s a very, very flattering thing, when somebody gets an erection in your company” (Graham, 74, gay). In ongoing relationships, participants found ways of communicating desire and pleasure through touch or talk. However, this was described as more difficult in the context of casual sex, where “if you’re not putting out all signs that you might be interested then people get the wrong message” (Euan, 67, gay), and “if you can’t get an erection at the sauna guys tend to turn away” (Cameron, 65, bisexual).

The consequence of ED for many participants was a sense of sexual incompetence in comparison with other gay men, particularly in the context of casual relationships. As Andy (61, gay) commented, “as a gay man and interacting with other gay men, yeah….I’d feel a little bit worthless.” David (64, gay) said that he tended “to withdraw somewhat when there is light-hearted banter between guys about their (sexual) experiences…because I can’t experience that anymore,” feeling “inadequate” as a result. Envy of other gay men who were not experiencing ED was also common. As Clive (70, gay) said “you look at other guys who are your age, but still active, and you think, ‘What about me? It isn’t fair. I’ve paid my money, I want my share’.”

At the same time, some participants reported an impact of ED within long-term relationships. For example, William (67, gay) said that his partner kept asking him “when are we going to have sex?,” and then started a relationship with another man when William could not perform, which made him feel like a “cuckold.” Zachary (59, gay, partner) described a deep sense of loss and sadness following his partner’s erectile changes, which impacted on their shared intimacy and pleasure:

Yes, there is an under-lying sadness that rides tandem to the joy of our coupling. We have fallen so far away from where we were. The intimacy we had worked at so hard was paying off big time as our love-making was so focused on each other—and then this. Now the impetuosity is gone, so too the erection at my back while sleeping, the kick from putting my hand on his cock and feeling a response, all changed by pills and timing. The intimacy has changed, now more focused on nurturing my partner’s emotional needs.

Zachary stands as an example of the many long-term partners who were described as “supportive and sympathetic,” “loving,” and “understanding,” resulting in a “feeling of security,” or “greater closeness in the relationship.” This closeness was sometimes associated with renegotiation of sexual activity in the face of ED, focusing on non-penetrative sex, such as oral sex, cuddling and stroking, use of sex toys, and frotting (rubbing genitals together). Many men described greater “intensity” in sexual connection with their partner as a result, as Terry (53, gay, partner) describes:

If you’d said to me prior to the operation that you would have felt the intensity of love and lovemaking that you felt in the first year, you know, new love and all that sort of stuff, I would have said you are bonkers, but that’s exactly what’s happened to us.

Other participants talked of enjoying “gentler and slower, more intimate, sort of, process” of “sexual play,” compared with the “aggressiveness” pre-cancer sex (Bruce, 61, gay). Medical aids were also described as allowing sex to continue post PCa, as Matt (56, gay, partner) said: “he’s woken up beforehand and got the injection ready and woken me up and away we go.” A minority of men talked of including an additional partner into a long-term relationship, or encouraging their partners to “play out” with other men, with positive benefits in terms of support and vicarious sexual pleasure. As Bruce (61, gay) said of his partner:

I recognize that he’s got physical needs and I don’t have a problem with that. And he comes home to me all the time, and in fact, shares part of his fantasy life with me anyway.

These accounts demonstrate the importance of relational context in sexual renegotiation, and potential differences between the impact of sexual changes in casual and long-term relationships.

### Anal Sensitivity and Changed Sexual Roles: “It’s a Very Sensitive Part of a Man’s Body”

Prior to PCa, 31 % of survey participants reported being insertive partners in anal intercourse (“tops”); 19 % were receptive partners (“bottoms”); 20 % enjoyed both (“versatile”); and 31 % did not engage in anal intercourse (survey items developed for this study). After cancer, 12 % of respondents described themselves as tops, 24 % as bottoms, 8 % as versatile, and 56 % had no anal intercourse at all. However, transitioning from being a top or versatile to a bottom was not an inevitable solution to erectile difficulty, as discomfort or pain during anal sex was reported by many men, as Sam (74, gay) described, in response to the suggestion from his psychologist that he could “change roles”: “No, I couldn’t. Because of my bowel problem and the radiation burn, I don’t think it’d be possible for me to do that.” Bruce (age 61, gay) described himself as having become “versatile,” but said that “in my recovery stage I had to be very careful about being a receptive partner because I found that could be quite painful.”

PCa treatment and removal of the prostate can also result in changes to anal sensitivity which can impact upon the sexual pleasure and satisfaction of men who were receptive partners before treatment. This is evident in the following accounts: “it’s a very sensitive part of a man’s body, and it is a great part of the enjoyment of anal sex…and so without them [prostate] a great deal of the enjoyment disappears” (Jack, 59, gay); “in terms of penetrative sex, when I’m the receiver, the pleasure that I had for that has basically gone” (Rick, 59). This suggests that some men may cease being receptive after treatment, due to lack of pleasure. Conversely, for a minority of men, anal sensitivity was described as having increased following PCa treatment, with Bruce (61, gay) suggesting that the “intense sexual gratification” provided by the prostate had masked other areas of sensitivity that he had “not necessarily realized or engaged” meaning “the simple act of being on the receptive end of sex is somehow more satisfying than it used to be.”

A number of men were reluctant to become the receptive partner because of what it meant to them in terms of sexual role, not wanting to take up what can be regarded by some men as a submissive position, or not finding it a pleasurable experience: “it doesn’t appeal to me at all” (Damon, 52, gay); “it was like an unevenness in the sexual relationship. The sex became more about the other person and their enjoyment of it and it was something I was almost doing just for them” (Mark, 45, gay). Having a regular partner who was normally a bottom, and difficulty in finding the right top, was also reported: “I’ll have to be the bottom but if your partner is a bottom as well, it’s not necessarily going to work” (Andy, 61, gay).

### Loss of Sexual Pleasure and Libido: “It’s a Profound Change in Identity”

When asked to rate their sexual desire in the last 4 weeks, 58 % of survey respondents rated it as absent, very poor, or fair, with level of sexual desire described as a problem by 65 % (EPIC). In the interviews, a number of men described sexual desire as “just not there” (Tony, 74, gay); or as having “no sexual drive or inclination whatsoever, like it’s been turned off, it’s really strange” (William, 67, gay). The absence of desire was reported to have a profound effect on identity. Andy (61, gay) described it as having “gone from being virile to not being virile” which felt as if “you’ve had a lot taken away from you.” Gordon (56, bisexual) expressed anger and frustration at the absence of libido and at attempts to resuscitate such feelings through medical aids:

It’s almost impossible to describe to someone who’s past the age of puberty what it’s like to feel no sexuality at all. It’s a profound change in identity and I can’t say that any more clearly and deeply and effectively. It really makes a huge difference. So when the question such as, “Well after you lost all libido did you try any of these aids?” It’s sort of like you’re asking a double amputee, “So did you try—?” “No, dear, I don’t have fingernails” [laughs]. It—that makes no sense.

The consequence of absence of desire, for some men, was sexual abstinence: “I don’t have sex”; “I can’t be bothered pursuing the idea.” For others, sex was enacted without desire, as a way of attempting to maintain sexual functioning:

My excursions out to [gay sex] venues these days are less to do with feeling horny and sexual, but more to do with…pumping a little bit of oxygen into my dick, just so that I won’t seize up altogether….There’s no love or sexuality involved in it anymore (Scott, 59, gay).

Scott did not report any pleasure in his sexual encounters, positioning them as “exercise.” He was not alone. Fifty percent of survey respondents reported experiencing little or no enjoyment during sex, compared with only four per cent of men rating sex as lacking enjoyment before cancer (CSFQ). Absence of pleasure during orgasm was the most commonly reported experience, with the ability to climax described as “frustrating” or “needing a lot of work.” Absence of ejaculation during orgasm was also reported to result in loss of pleasure, as well as changing the meaning of sexual encounters because of the absence of semen, outlined below.

### Non-Ejaculatory Orgasms: Loss of “An Essential Part of Sexual Enjoyment to Both Partners”

Seventy-one percent of survey participants reported complete loss of ejaculation at orgasm following PCa treatment, with an additional 13 % of men reporting that they ejaculated “rarely” or “sometimes” (CSFQ). Fifty-two percent of survey participants reported being “somewhat” (21 %) or “very” (31 %) concerned about their ability to ejaculate (CSFQ). Many men gave accounts of loss of sensation and pleasure as a result of ejaculatory loss: “climax doesn’t feel complete without the feeling of ejaculation”; “I don’t ejaculate any more. I never will. I miss it a great deal.” For some men, the magnitude of this loss was unforeseen: “lack of semen has affected me much more than I expected…it’s more difficult to talk about than erection issues” (Greg, 53, gay).

The absence of semen in sexual encounters, and the potential effect on partners, was reported to be a major concern. Ejaculation of semen stands as visible evidence of sexual satisfaction and excitement, as Clive (70, gay) commented, “ejaculation is an essential part of sexual enjoyment to both partners.” The socially validated nature of external semen exchange was emphasized in Henry’s description of a safe-sex publicity campaign “20, 30 years ago in the gay community in this country about ‘cum on him, not in him’,” which was aimed at encouraging men to avoid ejaculation in a partner in either anal or oral sex. Henry (59, gay) described the loss of this eroticized practice following PCa as a matter of “significance.” Absence of ejaculate was also associated with partner disappointment, as evidenced in the following accounts: “happy not to clean up. Not happy with partner’s disappointment” (Michael, 69, gay); “I miss the sensation of ejaculating and I think it disappoints my male partner” (Boris, 68, bisexual). Other men were concerned about disappointing future partners if they could not provide the “gift” of semen: “Semen is important to some prospective partners, this has restricted the number of potential partners” (Greg, 53, gay); “I miss the sensation of pumping ejaculate. I am also concerned that some guys really enjoy swallowing a load or being ejaculated on and will be disappointed when I cannot provide that” (Arnold, 57, gay). These concerns were borne out in the accounts of a number of partners we interviewed, who described missing the visible evidence of pleasure signified by ejaculation. This is illustrated in Anton’s account:

when you ejaculate you watch someone’s face and you hear the noises they make, you know that they are effectively engaged in that process and enjoying it to a degree, whereas when that’s not present it makes it a little bit more unknown (Anton, 54, gay, partner).

The consequence was that many men worried that they would be judged as a failure as a result of ejaculatory absence: “my fear is that they think less of me. Ah, in the fact that I can no longer ejaculate” (Lucian, age 51, gay); “I worry in my mind that I’m judged that I haven’t been enjoying the other person” (Mason, 68, gay). This resulted in avoidance of casual sex, where the absence of semen, often combined with erectile difficulties, would have to be explained: “it would be too hard to kind of disclose or to pick up somebody and say, ‘well, nothing is going to happen on my part, you know…. I can’t cum’” (Andy, 61, gay). The solution for some men was to take on the role of “top,” as “the lack of ejaculate is of little concern when you cannot see this not occurring” (Jack, 59, gay), but this requires confidence in still having the capacity for a firm erection. Other men reported achieving vicarious pleasure through a partner’s ejaculation: “I’m still enjoying giving my male partner oral sex because I get to enjoy his ejaculations vicariously” (Boris, 68, bisexual).

### Urinary Incontinence and Climacturia: “You Lose Your Body Management”

Sixty-five percent of survey participants reported changes in urinary patterns, primarily urinating more often following PCa, with 40 % reporting that problems with urinating limited their activities, and 25 % saying that they had difficulties urinating (FACT-P). In the open-ended survey items and interviews, men focused on the implications of urinary incontinence in the sexual and social arena. Many men reported climacturia, urinating during climax, as Pete (73, gay) commented: “It comes out, about the normal time of having an orgasm, but it just comes out in high pressure wee, instead of the normal white stuff.” Others reported urinary leakage during arousal or anal sex: Clive (70, gay) said that “when you get a bit excited you tend to leak a bit. You seem to lose your body management a bit”; and Lucian (51, gay) reported:

Due to the fact that I’m still slightly incontinent having anal sex is almost impossible as you have to relax, with the consequence of leakage, and even masturbation is difficult as I would leak sometimes quite a lot. So rather than be embarrassed I no longer have sex.

Lucian was not alone in avoiding sex. Many participants reported avoidance of casual sex as explaining climacturia was “too difficult,” “unsexy,” “humiliating,” or “embarrassing.” Negative reactions from prospective casual partners who were being informed of potential leakage of urine or blood were common, with Gordon (56, bisexual) explaining that when he met men on-line he would say “when I climax there’s usually some spurting of urine,” which acted as a “turn-off,” and sex would not happen. Avoidance of sex with a regular or long-term partner was also reported, due to the practical difficulties of negotiating the consequences of urinary leakage during sex:

I just had to put up with being incontinent for three years and wear pads and all that kind of thing, so in terms of sexual activity, you can imagine it’s extremely limited….I’d finish up very wet and I’d have to have towels all over the bed, and, you know, hardly worth doing, basically (Morris, 74, gay).

### Reduction in Penis Size: “It’s a Blow to the Ego”

Many participants reported significant reductions in penis size following radical prostectomy or radiation treatment, going from what “a normal 6½, 7 inch penis” to “2–3 inches…literally, a couple of fingers and the thumb” (Gareth, 65, gay); losing “about half the length and half the diameter” (Mark, 45, gay); or having a penis that was “like in fantastically cold weather and it’s like that all the time” (Stanley, 78, gay), or was “slowly but surely disappearing…it’s not long before I’ll have a string on the end of it to find it to go to the toilet” (Pete, 73, gay). These changes were described as “bloody terrible,” “a blow to the ego,” “the most dramatic thing” to follow treatment, or a cause of suicidal ideation: “I would like to know the statistics of the suicides for guys, because, generally, the adjustment is absolutely mind blowing…because your dick shrinks and your diameter diminishes” (Drew, 64, gay).

Visibility and comparison of penis size between gay men, linked to negative consequences of penis size reduction, was evident in many accounts. For example, Scott (59, gay) said that “for a gay male, you know, we notice things like (loss of penis size). And other people do too.” Drew described comparing himself to his friends:

[I felt] bloody terrible, because I’ve always had a fairly decent dick…and a couple of our friends have got small dicks, so I thought, I’ve always thought, “you poor bastards,” and now I’m in the same boat as them.

Euan (66, gay) described the “shame” of walking around naked in the sauna: “you’ve got this bloody now little dick, it’s awful.” However, it was in the realm of sexual relationships that reduction in penis size was reported to have had the greatest impact. Mark (45, gay) described “losing the positive commentary” as his penis had previously been “a fair bit bigger than average,” which “was always a bit of a talking point when I had sex.” Scott (59, gay) said that “people used to be attracted to me” because of penis length, and that it was a “calling card” that has now “gone.” Cameron (65, bisexual) described being embarrassed about the fact that his penis was “often drawn right back into” his body, saying that “if I go into a relationship with someone I will have to say, ‘well, look, honestly, it used to be bigger than this’ [laughter].” These accounts demonstrate the negative meanings ascribed to real changes to the penis, in terms of self, gay identity, and sexual relationships.

### Heath Care Professional Support for Gay Sexual Concerns: “We’re Usually not Considered”

Eighty percent of survey respondents reported having discussed PCa-related sexual changes with a health care professional (HCP), in a survey item developed for this study. In the open-ended survey responses and interviews, a majority of participants expressed dissatisfaction with the level of information received. HCP discomfort in discussing sexuality was a common report, as evidenced in Jim’s (64, gay) account, “my health care providers seemed more uncomfortable than me to discuss prostate cancer and sex.” For many men, absence of HCP knowledge of the impact of PCa on gay sex was a concern, with anal sex, reduction in penis size, the prostate as a site of pleasure, and absence of ejaculate, mentioned as areas where information had been sought, but was not forthcoming. The dynamics of GB relationships were also often “not considered” by HCPs, who made the assumption that patients are heterosexual:

Most health care professionals and others working in the prostate cancer field have no understanding of the different ways that prostate cancer can affect gay and bisexual men. Not just sexually, but in the non-sexual side of relationships. It’s as though we’re invisible. (Henry, 59, gay)

Even if gay sex was addressed, the specific concerns of bisexual men were described as being neglected, as Bill (age 65, bisexual) commented “people understand Gay and Str8 but Bi guys don’t fit so we seem to be ignored. It tears you apart internally and we get no help.” A number of participants reported being met with negative responses when they attempted to discuss the specifics of gay sex with HCPs. For example, Gareth (65, gay) reported that he asked his doctor about reduction in penis size, which had stopped him from having sex, and his doctor replied “I don’t want to know anything about your sex life,” which Gareth concluded “was because I was gay.” This resulted in participants feeling dissatisfied with their treatment, and having to obtain information about sexual changes elsewhere.

In contrast, other participants gave positive accounts of HCP interactions, which were described as “very good and very helpful” (Sam, 74, gay), as a result of having a HCP who “understands and is completely comfortable talking about gay male sexuality” (Scott, 59, gay), and which lead to the patient feeling “completely looked after” (Timothy, 65, gay). Many participants emphasized the importance of HCP education about the differences between gay and heterosexual men, to ensure that these positive experiences would be universal: “[HCPs] should be made aware that issues pertaining to GBT men are quite different to heterosexual men” (Clive, 70, gay); “I think all urologists need educating about the differences between gay and straight sex” (Graham, 74, gay).

## Discussion

This study has demonstrated that while GB men report many of the same changes to their sexuality after PCa reported by heterosexual men—including ED, reduction in penis size, loss of libido, and non-ejaculatory orgasms—there are a number of GB-specific meanings ascribed to such changes that need to be understood, within the context of the construction of gay sex and gay identity, and GB men’s responses to sexual changes.

### Sexual Function

The magnitude of loss of erectile functioning, and ability to engage in penetrative sex across the sample, was comparable to rates reported in previous population studies of men with PCa (Penson et al., 2008; Smith et al., 2009). However, the rate of distress associated with ED was substantially higher than that reported in heterosexual men of a comparable age (Roberts, Lepore, Hanlon, & Helgeson, 2010), which confirms previous reports of significantly higher rates of psychological distress in GB men with PCa, associated with sexual changes (Hart et al., 2014; Ussher et al., 2016). This could be explained by the finding that men who engage in more frequent sexual activity report significantly lower ability to live with ED (Sommers et al., 2008), as more frequent sexual activity is found in population studies of gay men (Pitts et al., 2006). The significance of an erect penis in gay sex also cannot be underestimated (Asencio et al., 2009), with erectile functioning previously reported to have a greater importance in the sexual lives of gay men in comparison with heterosexual men (Bancroft, Carnes, Janssen, Goodrich, & Long, 2005). The ability to maintain an erection and perform coital sex has been described as the essence of the male role (Tiefer, 1994), with boys learning early in life that their “manhood is tied to their penis” (Zilbergeld, 1992, p. 32), a phallocentric conceptualization of masculinity that is also adopted by gay men (McInnes, Bradley, & Prestage, 2009). While previous research has recognized the impact of PCa on masculinity (Bokhour et al., 2001), feelings of relative lack and inadequacy may be greater for gay men because their partners are sexually functioning men with whom they can compare themselves (Fergus et al., 2002) and for whom they seek to provide sexual pleasure and satisfaction, in part signified by erection and ejaculation. Gay masculinity is already marginalized in relation to hegemonic masculinity, with gay men often not considered to be “real men” (Nardi, 2000), and gay masculinity standing as “the repository of whatever is symbolically expelled from hegemonic masculinity” (Connell, 1995, p. 78). This means that for gay men living with ED and other difficulties in sex following PCa, their already marginalized masculinity may take another blow, through the loss of ability to affirm the self through contact within a sexual community, one where they were among equals or peers as men, resulting in a challenge to both masculine and gay identity. In this vein, our findings refute the prediction that gay men would be more able to come to terms with challenges to their masculinity following PCa (Asencio et al., 2009).

Men who were able to reconcile themselves to sexual changes, incorporate such changes into their identity as gay men, or to enjoy alternative sexual practices, were less likely to report a challenge to gay identity following PCa-related ED. Threat of sexual disqualification that resulted from ED was particularly acute in casual relationships, where “flexible” (Barsky, Friedman, & Rosen, 2006) or “renegotiated” (Ussher, Perz, Gilbert, Wong, & Hobbs, 2013a) sexual practices were difficult to discuss or establish, and rejection by prospective partners, accompanied by embarrassment or shame on the part of the man with PCa, were anticipated. As gay men are more likely to engage in casual sexual relationships (Blank, 2005), or to have concurrent partners (Lyons & Hosking, 2014), this is likely to be a greater concern, compared with heterosexual men. Other research has shown that the majority of GB men are versatile in terms of sexual roles during anal intercourse (Lyons et al., 2011). This suggests that pursuing flexibility in sexual roles following PCa-induced ED may offer further sexual options (Dowsett, Lyons, Duncan, & Wassersug, 2014) and assuage some experiences of inadequacy and distress. However, secondary self-labeling in relation to preferences in sexual roles during anal intercourse is an important aspect of identity for other GB men (Wei & Raymond, 2011), and changing sexual roles is not always possible or desirable (Asencio et al., 2009; Moskowitz, Rieger, & Roloff, 2008). In addition, as the prostate is a pleasure center for gay men (Filiault et al., 2008), loss of pleasure or discomfort during anal sex following PCa may be a further deterrent to men engaging in the receptive role in anal intercourse, regardless of their preferred sexual role before PCa. Thus, while men may have the “physiological capacity to both penetrate and be penetrated (through anal intercourse)” (Moskowitz & Hart, 2011, p. 835), the corporeality of the body, as well as the discursive meanings attributed to anal sexual roles, will determine whether GB men continue to engage in anal intercourse, or whether they change anal sexual roles, or focus on other sexual practices, after PCa.

### Broader Effects on Sexual Life

ED is common in older men (Johannes et al., 2000); however, PCa can result in the sudden experience of ED, in addition to other sexual changes. In the present study, in addition to feelings of failure and inadequacy, ED signified aging, a self-positioning that has extremely negative connotations within gay male culture, where youthfulness and sexual functionality are highly valued (Martins, Tiggemann, & Churchett, 2008). While heterosexual men also express concerns about aging following PCa (Oliffe, 2005), it has been argued that gay men live in a culture that is particularly sexually objectifying (Martins, Tiggemann, & Kirkbride, 2007), resulting in many gay men experiencing “accelerated aging” (Slevin & Linneman, 2010), where they are deemed older at a younger age than heterosexual men might be. Sex is the primary domain within which bodily based decline and distress over such changes is experienced (Lodge & Umberson, 2013), which explains why the maintenance of erectile functioning through the use of medical aids allowed some GB men to position themselves as youthful, with an expectation of continuing sexual relationships into later life. Accounts of continued sexual interest and activity in older men, even if medically aided, also serves to challenge negative cultural discourses about sex and aging (Watters & Boyd, 2009) and the inevitability of being a lonely older gay man (Leonard, Duncan, & Barrett, 2013).

Cultural ideals of masculinity and youth are also associated with bodily control (Lodge & Umberson, 2013), with urinary incontinence potentially disrupting a sense of control in sexual activity for men with PCa. Previous research has reported that urinary incontinence (Punnen et al., 2013) and climacturia (Abouassaly, Lane, Lakin, Klein, & Gill, 2006) are associated with distress in men with PCa and that for some men urinary incontinence is worse than ED (Fergus et al., 2002). Our finding that difficulty in negotiating climacturia with casual or new partners was of primary concern suggests that this is a difficulty that might affect a substantial proportion of GB men with PCa, given the open nature of many GB relationships. The absence of libido and sexual pleasure can also potentially disrupt a sense of sexual confidence and competence for men with PCa (Burns & Mahalik, 2007), with an additional threat to gay identities, due to the centrality of sex to GB masculinity (Nardi, 2000). For GB men, this loss of pleasure was accentuated by the absence of ejaculate during orgasm. It has previously been reported that most heterosexual men with PCa “are not bothered by absence of ejaculate,” but that it may interfere with sexual satisfaction (Benson, Serefoglu, & Hellstrom, 2012, p. 1149). In addition to loss of sexual pleasure during non-ejaculatory orgasms, gay men with PCa also grieve the absence of the ejaculate itself (Mitteldorf, 2005), as semen is of erotic significance during gay male sex (Prestage, Hurley, & Brown, 2013), and exchange of semen is a central objective of sex for some GB men (Holmes & Warner, 2005). Exchange or “gifting” (Holmes & Warner, 2005) of semen signifies intimacy and connection with partners (Schilder et al., 2008), resulting in partner disappointment at absence of ejaculate, providing explanation for previous reports that gay men report higher rates of ejaculatory bother after PCa than heterosexual men (Ussher et al., 2016; Wassersug et al., 2013).

Reduction in penis size has been reported as a concern for many heterosexual men treated for PCa (Parekh et al., 2013; Powel & Clark, 2005). There are further issues in regard to penis size for GB men (Thomas, Wootten, & Robinson, 2013). In gay male culture, the size of a man’s penis signifies sexual attractiveness and sexual viability, with penises “seen, compared, (and) contrasted” (Drummond & Filiault, 2007, p. 124), and a below-average-sized penis associated with lower psychosocial adjustment (Grov, Parsons, & Bimbi, 2010). In contrast, a large penis is representative of heightened masculinity within gay male culture (Drummond & Filiault, 2007), resulting in potential emasculation in the social domain following PCa, as evidenced by participant accounts in the present study. These concerns about reduced sexual desirability associated with penis size are not unfounded. Previous research has reported that gay men have a preference for partner with a large penis (Moskowitz, Rieger, & Seal, 2009), with smaller penis sizes linked to sexual dissatisfaction due to being “boring” or not being able to be “felt,” meaning that “in a gay world, the bigger the dick usually the more people want to have sex with you” (Drummond & Filiault, 2007, p. 125). Penis size is also associated with men’s sexual roles in anal sex, with men who have smaller penises more likely to identify as bottoms (Grov et al., 2010; Moskowitz & Hart, 2011). This suggests that change in penis size after PCa surgery may also impact upon GB sexual roles, encouraging men to take up a receptive role in anal sex.

### Provision of Health Care Information

The findings of the present study support previous reports of patient and partner dissatisfaction with HCP information provision about sexual changes experienced after PCa (Gilbert, Perz, & Ussher, 2014; Kelly, Forbat, Marshall-Lucette, & White, 2015). This has been associated with lack of confidence, training, or knowledge about sexuality after cancer on the part of HCPs; limitations of the clinical setting; and HCP avoidance of sexuality discussion with older patients, those from culturally and linguistically diverse (CALD) communities, and those who are LGBT (Hordern & Street, 2007; Ussher et al., 2013b). Gay men with PCa have also reported greater dissatisfaction with health care in comparison with heterosexual men (Torbit et al., 2015; Ussher et al., 2016), as well as difficulties related to heteronormative health information (Blank, 2005; Rose et al., 2016). These findings, combined with accounts of participants in the present study, reinforce the need for education and training of HCPs in the specific needs of GB men with PCa, as well as the development of targeted GB programs of supportive intervention (Buchting et al., 2015).

### Study Strengths and Limitations

The strengths of this study include the mixed method approach, which facilitated examination of sexual changes after PCa across a substantive sample of GB men through standardized measures, as well as in-depth analysis of the subjective experience of such changes through interviews of a sub-sample. Both the survey and interview samples stand as the largest number of GB men with PCa, and male partners of such men, researched quantitatively and qualitatively to date, addressing calls for research on this previously neglected population (Filiault et al., 2008). The limitations of the study include the use of a highly educated volunteer sample, which may not be representative of all GB men with PCa; the use of multiple methods of recruitment that does not allow for calculation of response rate; and initial participation through completion of an on-line survey, which may attract participants who have treatment side effects, or for whom sexual changes are important. Future research should ideally recruit through cancer registries or clinical contexts; however, this is difficult at present, as information on sexual orientation data is not routinely collected by cancer registries (Quinn et al., 2015), and clinics focusing on GB men with PCa are rare. Bisexual men and male partners also made up a relatively small proportion of the sample, despite concerted efforts to recruit such men, suggesting further research is needed in this area. We did not ask about HIV status, or about experiences participants may have had in relation to HIV, which may have impacted upon their mode of coping with PCa; future research should examine this issue. Finally, it would be useful to compare the experiences of GB men with PCa with GB men who have other types of cancer (sexual and non-sexual), in order to elucidate factors that are specific to GB men across cancers.

### Conclusion

This study has demonstrated that while GB men experience the same sexual changes after PCa that have been reported by heterosexual men, there are a number of GB-specific meanings and psychological consequences attached to sexual changes that need to be considered by researchers and clinicians, in the context of the discursive construction of gay sex and gay identity. When designing studies to examine the impact of PCa on men’s sexuality and quality of life, researchers need to ask about sexual orientation, and include questions on anal sex, ejaculatory bother, climacturia, and reduction in penis size—concerns that are often overlooked. Equally, when clinicians are advising men of the sexual consequences of PCa treatment, they need to provide information and support relating to the broad spectrum of sexual changes, in addition to information on ED. Clinicians also need to be aware of the specific meaning of sexual changes for GB men, in the context of both long-term and casual sexual relationships, and to avoid heteronormative assumptions about their patients. Only then will we be able to address the concerns and needs of the hitherto “hidden population” of GB men with PCa.

## References

[CR1] Abouassaly R, Lane BR, Lakin MM, Klein EA, Gill IS (2006). Ejaculatory urine incontinence after radical prostatectomy. Urology.

[CR2] Arrington MI (2003). “I don’t want to be an artificial man”: Narrative reconstruction of sexuality among prostate cancer survivors. Sexuality and Culture.

[CR3] Asencio M, Blank T, Descartes L, Crawford A (2009). The prospect of prostate cancer: A challenge for gay men’s sexualities as they age. Sexuality Research and Social Policy.

[CR4] Australian Institute of Health and Welfare (2012). Australia’s health 2012.

[CR5] Australian Institute of Health and Welfare. (2015). *Cancer in Australia: An overview 2014* (Vol. Cancer series No. 90 [Internet]. Cat. no. CAN 88). Canberra: AIHW.

[CR6] Bancroft J, Carnes L, Janssen E, Goodrich D, Long J (2005). Erectile and ejaculatory problems in gay and heterosexual men. Archives of Sexual Behavior.

[CR7] Barsky J, Friedman M, Rosen R (2006). Sexual dysfunction and chronic illness: The role of flexibility in coping. Journal of Sex and Marital Therapy.

[CR8] Benson CR, Serefoglu EC, Hellstrom WJG (2012). Sexual dysfunction following radical prostatectomy. Journal of Andrology.

[CR9] Blank TO (2005). Gay men and prostate cancer: Invisible diversity. Journal of Clinical Oncology.

[CR10] Bokhour BG, Clark JA, Inui TS, Silliman RA, Talcott JA (2001). Sexuality after treatment for early prostate cancer: Exploring the meanings of “erectile dysfunction”. Journal of General Internal Medicine.

[CR11] Braun V, Clarke B (2006). Using thematic analysis in psychology. Qualitative Research in Psychology.

[CR12] Buchting, F. O., Margolies, L., Bare, M. G., Bruessow, D., Díaz-Toro, E. C., Kamen, C., … Scout. (2015). *LGBT best and promising practices throughout the cancer continuum*. Fort Lauderdale, FL: LGBT HealthLink. Available online at http://www.lgbthealthlink.org/.

[CR13] Burns SM, Mahalik JR (2007). Understanding how masculine gender scripts may contribute to men’s adjustment following treatment for prostate cancer. American Journal of Men’s Health.

[CR14] Cancer Research UK. (2015). *Cancer survival for common cancers*. Retrieved 4th September, 2015 from http://www.cancerresearchuk.org/health-professional/cancer-statistics/survival/common-cancers-compared#heading-One.

[CR15] Chung E, Brock G (2013). Sexual rehabilitation and cancer survivorship: A state of art review of current literature and management strategies in male sexual dysfunction among prostate cancer survivors. Journal of Sexual Medicine.

[CR16] Connell RW (1995). Masculinities.

[CR17] Daniel, A., & Haddow, S. (2011, March). Erectile dysfunction after prostate cancer. *The Clinical Advisor*, pp. 64–68.

[CR18] Dowsett GW, Lyons A, Duncan D, Wassersug RJ (2014). Flexibility in men’s sexual practices in response to iatrogenic erectile dysfunction after prostate cancer treatment. Sexual Medicine.

[CR19] Drummond M, Filiault S (2007). The long and the short of it: Gay men’s perception of penis size. Gay & Lesbian Issues and Psychology Review.

[CR20] Duncan D, Watson J, Westle A, Mitchell A, Dowsett G (2011). Gay men and prostate cancer: Report on an audit of existing resources and websites providing information to men living with prostate cancer in Australia.

[CR21] Esper P, Mo F, Chodak G, Sinner M, Cella D, Pienta KJ (1997). Measuring quality of life in men with prostate cancer using the functional assessment of cancer therapy prostate instrument. Urology.

[CR22] Fergus KD, Gray RE, Fitch MI (2002). Sexual dysfunction and the preservation of manhood: Experiences of men with prostate cancer. Journal of Health Psychology.

[CR23] Filiault SM, Drummond MJ, Riggs DW (2009). Speaking out on GBT men’s health: A critique of the Australian government’s Men’s Health Policy. Journal of Men’s Health.

[CR24] Filiault SM, Drummond MJN, Smith JA (2008). Gay men and prostate cancer: Voicing the concerns of a hidden population. Journal of Men’s Health.

[CR25] Galbraith ME, Crighton F (2008). Alterations of sexual function in men with cancer. Seminars in Oncology Nursing.

[CR26] Gilbert E, Perz J, Ussher JM (2014). Talking about sex with health professionals: The experience of people with cancer and their partners. European Journal of Cancer Care.

[CR27] Gilbert E, Ussher JM, Perz J, Wong WKT, Hobbs K, Mason C (2013). Men’s experiences of sexuality after cancer: A material discursive intra-psychic approach. Culture, Health & Sexuality.

[CR28] Grov C, Parsons JT, Bimbi DS (2010). The association between penis size and sexual health among men who have sex with men. Archives of Sexual Behavior.

[CR29] Hart TL, Coon DW, Kowalkowski MA, Zhang K, Hersom JI, Goltz HH, Latini DM (2014). Changes in sexual roles and quality of life for gay men after prostate cancer: Challenges for sexual health providers. Journal of Sexual Medicine.

[CR30] Hartman M-E, Irvine J, Currie KL, Ritvo P, Trachtenberg L, Louis A, Matthew AG (2014). Exploring gay couples’ experience with sexual dysfunction after radical prostatectomy: A qualitative study. Journal of Sex and Marital Therapy.

[CR31] Holmes D, Warner D (2005). The anatomy of a forbidden desire: Men, penetration and semen exchange. Nursing Inquiry.

[CR32] Hordern AJ, Street AF (2007). Constructions of sexuality and intimacy after cancer: Patient and health professional perspectives. Social Science and Medicine.

[CR33] Johannes CB, Araujo AB, Feldman HA, Derby CA, Kleinman KP, McKinlay JB (2000). Incidence of erectile dysfunction in men 40 to 69 years old: Longitudinal results from the Massachusetts male aging study. Journal of Urology.

[CR34] Keller A, McGarvey EL, Clayton AH (2006). Reliability and construct validity of the changes in Sexual Functioning Questionnaire Short-Form (CSFQ-14). Journal of Sex and Marital Therapy.

[CR35] Kelly D, Forbat L, Marshall-Lucette S, White I (2015). Co-constructing sexual recovery after prostate cancer: A qualitative study with couples. Translational Andrology and Urology.

[CR36] Lee TK, Breau RH, Eapen L (2013). Pilot study on quality of life and sexual function in men-who-have-sex-with-men treated for prostate cancer. Journal of Sexual Medicine.

[CR37] Leonard W, Duncan D, Barrett C, Kampf A, Marshall BL, Petersen AR (2013). What a difference a gay makes: The constitution of ‘older gay men’. Aging men, masculinities and modern medicine.

[CR38] Liau A, Millett G, Marks G (2006). Meta-analytic examination of online sex-seeking and sexual risk behavior among men who have sex with men. Sexually Transmitted Diseases.

[CR39] Lodge AC, Umberson D (2013). Age and embodied masculinities: Midlife gay and heterosexual men talk about their bodies. Journal of Aging Studies.

[CR40] Lyons A, Hosking W (2014). Prevalence and correlates of sexual partner concurrency among Australian gay men aged 18–39 years. AIDS and Behavior.

[CR41] Lyons A, Pitts M, Smith G, Grierson J, Smith A, McNally S, Couch M (2011). Versatility and HIV vulnerability: Investigating the proportion of Australian gay men having both insertive and receptive anal intercourse. Journal of Sexual Medicine.

[CR42] Martins Y, Tiggemann M, Churchett L (2008). The shape of things to come: Gay men’s satisfaction with specific body parts. Psychology of Men & Masculinity.

[CR43] Martins Y, Tiggemann M, Kirkbride A (2007). Those speedos become them: The role of self-objectification in gay and heterosexual men’s body image. Personality and Social Psychology Bulletin.

[CR44] McInnes D, Bradley J, Prestage G (2009). The discourse of gay men’s group sex: The importance of masculinity. Culture, Health & Sexuality.

[CR45] McNair RP, Hegarty K (2010). Guidelines for the primary care of lesbian, gay, and bisexual people: A systematic review. Annals of Family Medicine.

[CR46] Mitteldorf D (2005). Psychotherapy with gay prostate cancer patients. Journal of Gay & Lesbian Psychotherapy.

[CR47] Moskowitz DA, Hart TA (2011). The influence of physical body traits and masculinity on anal sex roles in gay and bisexual men. Archives of Sexual Behavior.

[CR48] Moskowitz DA, Rieger G, Roloff ME (2008). Tops, bottoms and versatiles. Sexual and Relationship Therapy.

[CR49] Moskowitz DA, Rieger G, Seal DW (2009). Narcissism, self-evaluations, and partner preferences among men who have sex with men. Personality and Individual Differences.

[CR50] Motofei IG, Rowland DL, Popa F, Kreienkamp D, Paunica S (2011). Preliminary study with bicalutamide in heterosexual and homosexual patients with prostate cancer: A possible implication of androgens in male homosexual arousal. BJU International.

[CR51] Nardi P (2000). Gay masculinities.

[CR52] Oliffe J (2005). Constructions of masculinity following prostatectomy-induced impotence. Social Science and Medicine.

[CR53] Parekh A, Chen M-H, Hoffman KE, Choueiri TK, Hu JC, Bennett CL, Nguyen PL (2013). Reduced penile size and treatment regret in men with recurrent prostate cancer after surgery, radiotherapy plus androgen deprivation, or radiotherapy alone. Urology.

[CR54] Penson DF, McLerran D, Feng Z, Li L, Albertsen PC, Gilliland FD, Stanford JL (2008). 5-Year urinary and sexual outcomes after radical prostatectomy: Results from the prostate cancer outcomes study. Journal of Urology.

[CR55] Perz J, Ussher JM, Gilbert E (2014). Feeling well and talking about sex: psycho-social predictors of sexual functioning after cancer. BMC Cancer.

[CR56] Pitts M, Smith A, Mitchell A, Patel S (2006). Private lives: A report on the health and wellbeing of GLBTI Australians.

[CR57] Powel LL, Clark JA (2005). The value of the marginalia as an adjunct to structured questionnaires: Experiences of men after prostate cancer surgery. Quality of Life Research.

[CR58] Prestage G, Hurley M, Brown G (2013). “Cum Play” among gay men. Archives of Sexual Behavior.

[CR59] Punnen S, Cowan JE, Dunn LB, Shumay DM, Carroll PR, Cooperberg MR (2013). A longitudinal study of anxiety, depression and distress as predictors of sexual and urinary quality of life in men with prostate cancer. BJU International.

[CR60] Quinn GP, Sanchez JA, Sutton SK, Vadaparampil ST, Nguyen GT, Green BL, Schabath MB (2015). Cancer and lesbian, gay, bisexual, transgender/transsexual, and queer/questioning (LGBTQ) populations. CA: A Cancer Journal of Clinicians.

[CR61] Roberts KJ, Lepore SI, Hanlon AL, Helgeson V (2010). Genitourinary functioning and depressive symptoms over time in younger versus older men treated for prostate cancer. Annals of Behavioral Medicine.

[CR62] Rose D, Ussher JM, Perz J (2016). Let’s talk about gay sex: Gay and bisexual men’s sexual communication with healthcare professionals after prostate cancer. European Journal of Cancer Care.

[CR63] Rubin HJ (2005). Qualitative interviewing: The art of hearing data.

[CR64] Schilder AJ, Orchard TR, Buchner CS, Miller ML, Fernandes KA, Hogg RS, Strathdee SA (2008). ‘It’s like the treasure’: Beliefs associated with semen among young HIV-positive and HIV-negative gay men. Culture, Health & Sexuality.

[CR65] Siegel RL, Miller KD, Jemal A (2015). Cancer statistics, 2015. CA: A Cancer Journal for Clinicians.

[CR66] Slevin KF, Linneman TJ (2010). Old gay men’s bodies and masculinities. Men and Masculinities.

[CR67] Smith, D. P., King, M. T., Egger, S., Berry, M. P., Stricker, P. D., Cozzi, P., … Armstrong, B. K. (2009). Quality of life three years after diagnosis of localised prostate cancer: Population based cohort study. *BMJ, 339*, b4817. 10.1136/bmj.b4817 PMC2784818 19945997

[CR68] Sommers BD, Beard CJ, D’Amico AV, Kaplan I, Richie JP, Zeckhauser RJ (2008). Predictors of patient preferences and treatment choices for localized prostate cancer. Cancer.

[CR69] Susman E (2011). Gay men face extra burden coping with prostatectomy. Oncology Times.

[CR70] Thomas C (2012). An analysis of postings on two prostate cancer discussion boards. Gay and Lesbian Issues and Psychology Review.

[CR71] Thomas C, Wootten A, Robinson P (2013). The experiences of gay and bisexual men diagnosed with prostate cancer: Results from an online focus group. European Journal of Cancer Care.

[CR72] Tiefer L (1994). The medicalization of impotence: Normalizing phallocentrism. Gender and Society.

[CR73] Torbit LA, Albiani JJ, Crangle CJ, Latini DM, Hart TL (2015). Fear of recurrence: The importance of self-efficacy and satisfaction with care in gay men with prostate cancer. Psycho-Oncology.

[CR74] Ussher JM, Perz J, Gilbert E, Wong WKT, Hobbs K (2013). Renegotiating sex after cancer: Resisting the coital imperative. Cancer Nursing.

[CR75] Ussher JM, Perz J, Gilbert E, Wong WKT, Mason C, Hobbs K, Kirsten L (2013). Talking about sex after cancer: A discourse analytic study of health care professional accounts of sexual communication with patients. Psychology & Health.

[CR76] Ussher JM, Perz J, Kellett A, Chambers SK, Latini D, Davis I (2016). Health related quality of life, psychological distress and sexual changes following prostate cancer: A comparison of gay and bisexual men with heterosexual men. Journal of Sexual Medicine.

[CR77] Wassersug RJ, Lyons A, Duncan D, Dowsett GW, Pitts M (2013). Diagnostic and outcome differences between heterosexual and nonheterosexual men treated for prostate cancer. Urology.

[CR78] Watters Y, Boyd TV (2009). Sexuality in later life: Opportunity for reflections for healthcare providers. Sexual and Relationship Therapy.

[CR79] Wei JT, Dunn RL, Litwin MS, Sandler HM, Sanda MG (2000). Development and validation of the Expanded Prostate Cancer Index Composite (EPIC) for comprehensive assessment of health-related quality of life in men with prostate cancer. Urology.

[CR80] Wei C, Raymond H (2011). Preference for and maintenance of anal sex roles among men who have sex with men: Sociodemographic and behavioral correlates. Archives of Sexual Behavior.

[CR81] Wittman D, Northouse L, Foley S, Gilbert S, Wood DP, Balon R, Montie JE (2009). The psychosocial aspects of sexual recovery after prostate cancer treatment. International Journal of Impotence Research.

[CR82] Wong WK, Lowe A, Dowsett GW, Duncan D, O’Keeffe D, Mitchell A (2005). Prostate cancer information needs of Australian gay and bisexual men.

[CR83] Zaider T, Manne S, Nelson C, Mulhall J, Kissane D (2012). Loss of masculine identity, marital affection, and sexual bother in men with localized prostate cancer. Journal of Sexual Medicine.

[CR84] Zilbergeld B (1992). The new male sexuality.

